# From molecular evolution to the school health room: the role of community-based public health nurses (helsesykepleiere) in one health communication on cross-species influenza transmission in Norway

**DOI:** 10.3389/fcimb.2026.1965045

**Published:** 2026-09-22

**Authors:** Jörn Klein

**Affiliations:** Department of Nursing and Health Sciences, Faculty of Health and Social Sciences, University of South-Eastern Norway, Porsgrunn, Norway

**Keywords:** avian influenza, cross-species transmission, H5N1, health literacy, helsesykepleier, nordic public health, one health, pandemic preparedness

## Abstract

Highly pathogenic avian influenza (HPAI) A(H5N1) clade 2.3.4.4b viruses have undergone an unprecedented expansion of host range since 2021, moving from wild birds and poultry into dairy cattle, domestic cats, and other mammals, with sporadic human infections in Europe and North America (Food and Agriculture Organization et al., 2025a; Rolfes et al., 2025). This expansion is underpinned by well-characterised molecular mechanisms of influenza A virus evolution — antigenic drift, gene segment reassortment, and receptor-binding adaptation — that shape the frequency and direction of cross-species spillover (McDonald et al., 2016; Steel and Lowen, 2017; Man et al., 2026). Norwegian wild-bird and mammalian surveillance data document recurrent incursions of clade 2.3.4.4b viruses, and international risk assessments continue to describe low, but non-zero, human risk concentrated among individuals with close animal contact (World Health Organization, 2025–2026; Ytrehus et al; European Food Safety Authority et al., 2025a). Translating this fast-moving virological and epidemiological evidence into everyday protective behavior for children, adolescents, and families remains an underdeveloped link in pandemic preparedness, compounded by documented gaps in adolescent One Health literacy (Zucca et al., 2021). This mini review synthesizes current literature on the molecular basis of influenza cross-species transmission, the Norwegian epidemiological and One Health governance context, and established child- and family-oriented risk-communication frameworks, to argue that Norway’s community-based public health nurses (helsesykepleiere) — who deliver near-universal, statutorily mandated contact with children and families through the helsestasjon- and skolehelsetjeneste — occupy a distinctive but underused position as One Health communication nodes (Helsedirektoratet, 2021). I outline a practical three-tier framework for translating zoonotic-influenza science into age-appropriate household guidance, critically appraise the feasibility, workload, training, and governance implications of this role, and identify priority gaps for future empirical and implementation research.

## 1 Introduction

Influenza A viruses remain among the most consequential zoonotic pathogens of the past century, with all major human pandemics traced to animal reservoirs, most often through reassortment of segmented viral genomes in avian or swine intermediate hosts (de Silva et al., 2012; Steel and Lowen, 2017). Since 2021, however, the epidemiology of highly pathogenic avian influenza (HPAI) A(H5N1) clade 2.3.4.4b has departed markedly from this historical pattern. Rather than remaining largely confined to wild birds and commercial poultry, clade 2.3.4.4b viruses have spread into an unprecedented range of mammalian species, including foxes, seals, mink, domestic cats, and, since March 2024, dairy cattle in the United States (Food and Agriculture Organization et al., 2025a; Kamel et al., 2025). Sporadic human infections have followed this mammalian expansion, and while sustained human-to-human transmission has not been documented, international risk assessments describe a dynamic and evolving situation that warrants continued vigilance (World Health Organization, 2025–2026; Rolfes et al., 2025).

This shift has practical consequences that extend well beyond veterinary and occupational medicine. Families that keep backyard poultry, own cats fed raw-food diets, or live near wild-bird habitats are now plausibly exposed populations, not merely bystanders to a distant agricultural problem (Centers for Disease Control and Prevention, 2025a; Vaughan et al., 2026). Yet the substantial and rapidly evolving virological literature on cross-species transmission mechanisms is rarely translated into a form that reaches children, adolescents, and parents at the point where behavior change actually occurs — in the home, the farmyard, or the veterinary clinic waiting room. In Norway, one existing but underused channel for this translation is the corps of community-based public health nurses (helsesykepleiere), who deliver statutorily mandated, near-universal contact with children and families through the child and youth health centers and school health service (Helsedirektoratet, 2021). This mini review synthesizes current literature on (i) the molecular mechanisms underlying influenza A virus cross-species transmission, (ii) the global and Norwegian epidemiological landscape, (iii) Norwegian One Health governance and pandemic-preparedness structures, and (iv) established risk-communication and health-literacy frameworks for children and families, in order to outline a practical framework through which public health nurses can function as One Health communication nodes.

Evidence for this mini review was identified through structured searches of PubMed/MEDLINE, Web of Science, and Google Scholar for English-language literature published between January 2020 and August 2026, using combinations of the terms ‘H5N1’, ‘avian influenza’, ‘clade 2.3.4.4b’, ‘cross-species transmission’, ‘reassortment’, ‘One Health’, ‘risk communication’, ‘health literacy’, ‘helsesykepleier’, and ‘public health nursing’, supplemented by hand-searched reference lists and direct searches of institutional sources (FHI, Norwegian Veterinary Institute, Mattilsynet, WHO, EFSA, and CDC) for current risk assessments and guidance. Given the breadth of the topic and the mini review format, this is a narrative rather than a systematic review, and no formal quality-appraisal tool was applied. The specific search strings and retrieval steps are documented in **Supplementary Search Record S1**.

## 2 Molecular evolution and mechanisms of cross-species transmission

Influenza A viruses possess a genome of eight discrete, single-stranded, negative-sense RNA segments, a structural feature that permits two distinct evolutionary pathways (McDonald et al., 2016). The first, antigenic drift, results from the gradual accumulation of point mutations introduced by the error-prone viral RNA polymerase during replication. The second, and epidemiologically more consequential for cross-species jumps, is reassortment: when two distinct influenza A viruses co-infect the same host cell, progeny virions can package a novel combination of the eight parental segments, generating genetically — and potentially phenotypically — distinct viruses in a single replication cycle (Steel and Lowen, 2017; Selective genome packaging mechanisms of influenza A viruses, 2021). Reassortment between avian, swine, and human lineages produced the reassortant strains responsible for the 1957, 1968, and 2009 pandemics, historically via swine acting as a permissive co-infection host, or ‘mixing vessel’ (de Silva et al., 2012).

A second determinant of host range is the receptor-binding specificity of the viral hemagglutinin protein. Avian-adapted viruses preferentially bind α2,3-linked sialic acid receptors predominant in the avian gut and respiratory tract, whereas mammalian-adapted viruses, including human seasonal strains, preferentially bind α2,6-linked receptors characteristic of the upper mammalian respiratory tract. Single amino acid substitutions in the hemagglutinin receptor-binding site, together with adaptive changes in the viral polymerase complex, can shift this binding preference and materially increase a virus’s capacity to infect and transmit within a new mammalian host (Man et al., 2026). Among these changes, PB2 substitutions such as E627K and D701N improve polymerase activity at mammalian upper-respiratory-tract temperatures and are recurrently detected in mammalian-adapted clade 2.3.4.4b genotypes, making them closely watched risk markers. Recent genotypes circulating in North American dairy cattle and companion animals, including B3.13 and D1.1, carry constellations of such adaptive markers, and ongoing genomic surveillance by joint WHO/FAO/WOAH assessment continues to track their evolution for signals of increased mammalian or human transmissibility (Food and Agriculture Organization et al., 2025a; Man et al., 2026). Because most reassortant or drift-variant genotypes carry fitness costs that limit onward spread, only a minority of spillover events establish sustained transmission chains in a new host — but the frequency of coinfection opportunities, and hence the statistical opportunity for a high-fitness variant to emerge, rises directly with the scale of the current panzootic. This technical surveillance information, while central to expert risk assessment, is rarely rendered in a form accessible to non-specialist audiences, a gap addressed later in this review.

These molecular determinants of host range and transmissibility are not simply of virological interest. Because receptor-binding adaptation, polymerase mutation, and reassortment are the specific mechanisms that determine whether a spillover event remains a dead-end infection or acquires the capacity for onward transmission, they define the biological rationale for the tiered, exposure-specific communication framework proposed in Section 7: household guidance calibrated to the animal-contact pathways — raw feeding, wild-bird contact, dairy exposure — most likely to provide the coinfection or exposure opportunities described above. Framing the practical framework in these molecular terms is intended to keep the proposed nursing role anchored to virological evidence rather than to a generic exhortation to be alert to ‘bird flu’.

## 3 Current epidemiological landscape: global panzootic and the Norwegian context

Since its emergence in Europe in 2020–2021, clade 2.3.4.4b has become globally dominant, spreading across Europe, Africa, the Americas, and, more recently, Antarctica, with unprecedented mortality events in wild bird and marine mammal populations (Centers for Disease Control and Prevention, 2025b; Kamel et al., 2025). The detection of the virus in United States dairy cattle in March 2024 marked a further expansion: as of mid-2025, outbreaks had been confirmed in more than 1,000 herds across multiple states, with clinical signs in cattle including decreased milk yield, abnormal milk consistency, and reduced feed intake (Food and Agriculture Organization et al., 2025a; Morel et al., 2026). Between March 2024 and May 2025, a total of approximately 70 human cases of HPAI A(H5) infection were reported in the United States: 41 following exposure to infected dairy cattle, 24 to commercial poultry, two to backyard poultry flocks, and three with undetermined exposure (Centers for Disease Control and Prevention, 2025c). Most infections presented as mild conjunctivitis, occasionally accompanied by fever or respiratory symptoms; a minority required hospitalization, and one death occurred in a person with underlying health conditions exposed to a backyard flock and wild birds (Rolfes et al., 2025). A first pediatric case with an undetermined exposure source was identified in California in late 2024 (Zhu et al., 2025).

Companion animals have emerged as an additional and household-relevant transmission pathway. Domestic cats appear highly susceptible to severe, often fatal, disease, typically following consumption of raw milk, raw pet food, or contact with infected wild birds; a global review identified more than 600 documented feline infections across 12 species and 18 countries since the emergence of clade 2.3.4.4b (CIDRAP, 2025; Sykes, 2025). In late 2024, a cluster investigation in Los Angeles County identified serologic evidence consistent with possible virus transmission from an infected domestic cat to a veterinary professional — one of 25 serosurveyed contacts among 139 potentially exposed persons, with no virus isolated from the human case — providing what may be the first, if unconfirmed, instance of cat-to-human zoonotic transmission for this clade (Vaughan et al., 2026). To date, no sustained human-to-human transmission of clade 2.3.4.4b viruses has been documented anywhere, and joint WHO/FAO/WOAH and European risk assessments continue to classify general population risk as low, with elevated risk restricted to those with direct occupational or household exposure to infected animals (World Health Organization, 2025–2026; European Commission / EFSA).

Within this global picture, Norway has not been spared. Norwegian Veterinary Institute wild-bird and mammalian surveillance programs document recurrent clade 2.3.4.4b detections, including spillover into Norwegian foxes and seals, consistent with broader European surveillance trends reported by the European Food Safety Authority and European Centre for Disease Prevention and Control (Ytrehus et al; European Food Safety Authority et al., 2025a). As elsewhere, the general population risk in Norway is assessed as low, with the principal exposure pathway concentrated among individuals with occupational or hobbyist contact with wild birds, backyard poultry, or other domestic animals (Food and Agriculture Organization et al., 2025a) — a demographic that includes a meaningful proportion of Norwegian households with children, particularly in rural and peri-urban districts.

## 4 One health governance and pandemic preparedness in Norway

Norway has formally embedded One Health principles — the recognition that human, animal, and environmental health are interdependent — into its national approach to zoonotic disease and pandemic preparedness. The Norwegian Institute of Public Health identifies zoonotic influenza explicitly as a priority One Health working area, alongside food safety and antimicrobial resistance, and coordinates closely with the Norwegian Veterinary Institute and Norwegian Food Safety Authority on surveillance and risk communication (Norwegian Institute of Public Health (FHI), 2020). This institutional commitment is further reflected in Norway’s National One Health Strategy against Antimicrobial Resistance 2024–2033, which establishes cross-sectoral governance mechanisms that could, in principle, extend to influenza preparedness (Norwegian Ministry of Health and Care Services), and in an independent country-profile assessment noting that Norway has integrated One Health principles into its broader pandemic preparedness and response framework through interdisciplinary task forces and improved cross-sector data sharing (BeReady4Pandemics, 2025).

Notably, however, these governance frameworks are articulated predominantly at the level of surveillance coordination and inter-agency data sharing among human, animal, and environmental health authorities. None of the reviewed strategy or preparedness documents specify a defined role for frontline, community-based health personnel in the direct translation of One Health surveillance findings into household-level guidance. This represents an implementation gap between governance-level One Health ambition and the last-mile communication needed to change everyday behavior — a gap this review argues the helsesykepleier workforce is structurally well placed to help close.

## 5 The helsesykepleier as a one health communication node

Helsesykepleiere are specialist public health nurses operating within Norway’s statutorily mandated child health and school health services (helsestasjons- og skolehelsetjenesten), providing near-universal, longitudinal contact with children and families from infancy through upper secondary school, encompassing routine health checks, vaccination programs, and structured health guidance for parents and, later, directly for adolescents (Helsedirektoratet, 2021). This combination of near-total population reach, repeated contact over many years, and an existing mandate for preventive health counselling distinguishes the helsesykepleier role from generic public health messaging: unlike a one-off media campaign, the relationship is relational and cumulative, allowing guidance to be tailored to a given family’s actual animal exposures — for example, backyard poultry keeping, pet ownership, or proximity to wild-bird habitats — rather than delivered as an undifferentiated general warning.

This structural advantage is particularly relevant given documented deficits in existing zoonotic-disease literacy among the very age group the school health service works. A multi-country school-based survey found that adolescents across several European and non-European settings had limited understanding of One Health concepts and zoonotic transmission routes, including basic knowledge of how diseases move between animals and humans (Zucca et al., 2021). The Swedish National Veterinary Institute’s initiative on communicating One Health concepts to children offers a template of age-appropriate content — explaining zoonotic transmission through concrete, everyday examples rather than abstract virological language — that could plausibly be adapted for delivery through Norwegian Child and youth health centers and school health service contacts (Swedish National Veterinary Institute (SVA)). Positioning helsesykepleiere as a communication node in this sense does not require them to become infectious-disease specialists; rather, it treats zoonotic-influenza guidance as a natural extension of the preventive counselling repertoire they already deliver on nutrition, vaccination, and injury prevention.

## 6 Risk communication principles for children, adolescents, and families

Any translation framework of this kind should be grounded in established risk-communication and health-literacy science rather than improvised messaging. The United States Centers for Disease Control and Prevention’s Crisis and Emergency Risk Communication (CERC) model, widely applied to communicating outbreak risk to children, articulates six core principles: being first to provide information, being right, being credible, expressing empathy, promoting action, and showing respect (Abrams et al., 2022). For younger audiences specifically, this literature emphasizes honest but reassuring framing, avoidance of fear-based messaging, and actionable, age-appropriate content — principles echoed in the World Health Organization’s own strategic communications framework, which stresses accessibility, timeliness, and transparency as preconditions for public trust (World Health Organization).

Complementing these crisis-communication principles, health literacy frameworks describe the underlying competencies audiences need to act on health information: the World Health Organization’s model organizes this into the capacities to access, understand, appraise, remember, and use health information (World Health Organization, 2022), while the CDC’s Health Literacy Action Plan explicitly identifies the integration of developmentally appropriate health and science content into education and health services from preschool through university as a distinct strategic goal (Centers for Disease Control and Prevention, 2024). A structured review of European risk-communication guidelines by the European Centre for Disease Prevention and Control identified seven recurring elements of effective frameworks — problem formulation, stakeholder involvement, communication itself, quantitative risk assessment, iteration and evaluation, and informed decision-making — and a complementary ECDC prevention framework argues that health literacy should be treated as a system-level enabler of disease prevention, not merely an individual attribute to be assessed in isolation (European Centre for Disease Prevention and Control; European Centre for Disease Prevention and Control, 2013). Finally, a tiered model of child engagement in outbreak contexts distinguishes child-friendly communication (Level 1), activities that encourage children to share perspectives and take specific protective actions (Level 2), and genuine child participation in decisions affecting them (Level 3) — a scaffold that, while developed in a different geographic context, offers a useful graduated framework for age-appropriate helsesykepleier-led communication (Key Considerations: Child Engagement in the Context of Disease Outbreaks. Social Science in Action, 2025). The CDC’s Access and Functional Needs Toolkit further underscores those channels — schools, daycare facilities, and pediatric health services — through which family-level risk communication is most effectively multiplied (Centers for Disease Control and Prevention), channels in which the Norwegian helsesykepleier role is already embedded.

## 7 Translating evidence into practice: toward a practical framework

Bringing together the molecular, epidemiological, governance, and communication literatures reviewed above, I propose a three-tier practical framework for helsesykepleier-delivered One Health communication on cross-species influenza transmission.

First, household-level behavioral guidance: concrete, low-literacy-burden advice such as hand hygiene after contact with wild birds, backyard poultry, or their environments; never feeding pets raw milk, raw meat, or raw pet-food diets, given documented associations between raw feeding and severe feline H5N1 infection; avoiding contact with sick or dead wild birds and mammals; and knowing how to report unusual wildlife mortality events to the Norwegian Veterinary Institute or Norwegian Food Safety Authority (Mattilsynet) (Ytrehus et al; Centers for Disease Control and Prevention, 2025a; Vaughan et al., 2026). Second, age-stratified explanatory content: parent-facing material at child health service contacts for households with young children, and directly adolescent-facing material within the school health service, adapted from existing child-oriented One Health communication resources such as the Swedish SVA materials and structured according to the WHO health-literacy competency ladder of access, understanding, appraisal, retention, and use (Swedish National Veterinary Institute (SVA); World Health Organization, 2022). Third, a feedback and surveillance-liaison function, in which helsesykepleiere serve as an additional, informal sentinel channel — flagging unusual animal-exposure histories or clusters of related family concerns back into existing One Health surveillance structures, complementing formal wildlife, veterinary, and public health surveillance rather than duplicating it (Ghai et al., 2022; Chou et al., 2025).

Operationalizing this framework would require, at minimum, short evidence-based briefing materials co-produced by microbiology and infection-prevention researchers together with school health service leadership, in a format analogous to the vaccination information sheets already used within the Child and youth health centers and school health service; integration of zoonotic-influenza content into the nursing curricula that train future helsesykepleiere at Norwegian institutions such as the University of South-Eastern Norway; and formal evaluation of comprehension, retention, and behavior change following implementation, rather than treating the intervention as self-evidently effective.

This proposal is a hypothesis for evaluation, not a ready-to-deploy protocol, and its feasibility depends on constraints that deserve explicit discussion. Helsesykepleiere already report high caseloads within statutorily mandated consultation schedules, and national workforce assessments describe recurrent staffing shortfalls in the service (Helsedirektoratet, 2021); any added communication task must therefore be brief and scripted rather than a freestanding responsibility. The training implication is correspondingly modest: helsesykepleiere need short, competency-based instruction in recognizing relevant exposure histories and using standardized messaging, not virology training. The proposed surveillance-liaison function also raises unresolved governance questions: any flagging of unusual exposure histories back into One Health surveillance structures needs clear rules on what may be shared, with whom, and under what confidentiality safeguards, since helsesykepleiere operate under the same duty of confidentiality as other health personnel. Finally, this informal sentinel role has real limits: without a defined sampling denominator it cannot generate incidence estimates, it risks false alarms from misattributed symptoms, and it must not substitute for formal veterinary and public-health surveillance.

## 8 Research and practice gaps

Several gaps constrain immediate implementation of the framework proposed above. First, no Norwegian-specific studies comparable to the multi-country adolescent One Health knowledge survey currently exist, leaving the baseline level of zoonotic-influenza literacy among Norwegian children, adolescents, and parents unknown (Zucca et al., 2021). Second, no published evaluation has assessed helsesykepleier-delivered zoonotic-risk communication materials for comprehension, acceptability, or downstream behavior change. Third, formal data-sharing pathways between veterinary and wildlife surveillance bodies (the Norwegian Veterinary Institute, Mattilsynet) and the primary/community health service communication pipeline remain underdeveloped, limiting the feedback-liaison function proposed in tier three above. Fourth, because the molecular risk landscape is not static — new mammalian-adaptive genotypes continue to be characterized by ongoing genomic surveillance — any communication materials will require a mechanism for periodic scientific review and revision rather than one-off production (Food and Agriculture Organization et al., 2025a; Man et al., 2026). Fifth, governance and confidentiality safeguards for any surveillance-liaison function remain untested, with no published protocol specifying what may be shared with veterinary or public-health authorities, or under what consent arrangements. Addressing these gaps will require co-design research involving helsesykepleiere, microbiologists, veterinary epidemiologists, and families themselves, to define communication formats that are scientifically accurate, developmentally appropriate, and free of unwarranted alarm.

## 9 Conclusion

The molecular mechanisms driving the expanding host range of HPAI A(H5N1) clade 2.3.4.4b are the subject of continuous, increasingly granular scientific characterization, and Norwegian wildlife and veterinary surveillance is generating an increasingly detailed picture of local risk. What remains comparatively underdeveloped is the corresponding public-facing translation infrastructure connecting this evolving evidence base to the households where behavior actually changes. Norway’s helsesykepleiere, by virtue of their statutory mandate and near-universal, longitudinal reach into families with children, constitute an existing but underused resource for this translation task — one consistent with, and potentially strengthening, Norway’s own stated One Health governance ambitions (Norwegian Ministry of Health and Care Services; BeReady4Pandemics, 2025). Realizing this potential will require deliberate interdisciplinary collaboration between infection-prevention and virology research groups and the school health service, together with the evaluation research needed to ensure that any resulting communication genuinely improves family-level understanding and protective behavior, rather than simply adding to an already crowded preventive-health agenda. Realizing this potential is conditional on the workload, training, and governance safeguards above being resourced, not assumed cost-free.
