# Supplementary material for: From molecular evolution to the school health room: the role of community-based public health nurses (helsesykepleiere) in one health communication on cross-species influenza transmission in Norway
Source: Front Cell Infect Microbiol. 2026 Sep 22;16:1965045. doi: 10.3389/fcimb.2026.1965045 (PMC13639862; doi:10.3389/fcimb.2026.1965045)
Supplement: Supplementary file 1 [file DataSheet1.docx]

## Supplementary Search Record S1

**Manuscript:** From Molecular Evolution to the School Health Room: The Role of Community-Based Public Health Nurses (Helsesykepleiere) in One Health Communication on Cross-Species Influenza Transmission in Norway

**Purpose:** This record documents the evidence-identification approach underlying the mini review, in response to reviewer comment 1 (clarify databases, search dates, keywords, and study-selection criteria). It supplements the methodology paragraph now included in the manuscript (immediately following the Introduction).

### 1. Review type

This is a narrative mini review, consistent with the Frontiers Mini Review article-type format. It is not a systematic review: no protocol was pre-registered, screening was not conducted by two independent reviewers against a documented denominator, and no formal quality-appraisal (risk-of-bias) tool was applied. This is stated explicitly in the manuscript’s methodology paragraph.

### 2. Databases and sources searched

| Source | Role |
| --- | --- |
| PubMed/MEDLINE | Primary biomedical literature database |
| Web of Science | Cross-disciplinary literature and citation searching |
| Google Scholar | Supplementary/grey-literature discovery |
| Hand-searching of reference lists | Identification of additional relevant sources cited within retrieved articles |
| Norwegian Institute of Public Health (FHI) | Direct search for national risk assessments and guidance |
| Norwegian Veterinary Institute | Direct search for wildlife/mammalian surveillance data |
| Norwegian Food Safety Authority (Mattilsynet) | Direct search for food-safety and animal-health guidance |
| World Health Organization (WHO) | Direct search for global risk assessments and communication frameworks |
| European Food Safety Authority (EFSA) / European Centre for Disease Prevention and Control (ECDC) | Direct search for European risk assessments and risk-communication guidance |
| United States Centers for Disease Control and Prevention (CDC) | Direct search for US surveillance summaries and case data |

### 3. Search window

English-language literature published between **January 2020 and August 2026**. This window was chosen to capture the period of accelerated clade 2.3.4.4b host-range expansion (from 2021 onward) while including foundational virological literature on influenza A reassortment and receptor-binding adaptation published slightly earlier.

### 4. Search terms and concept groups

Searches combined terms from the following concept groups (used in various Boolean combinations appropriate to each database’s syntax):

- **Virological/molecular:** “H5N1”, “avian influenza”, “clade 2.3.4.4b”, “cross-species transmission”, “reassortment”, “receptor binding”, “polymerase adaptation”, “PB2”
- **Epidemiological/surveillance:** “human cases”, “dairy cattle”, “domestic cats”, “wild birds”, “serologic evidence”, “One Health surveillance”
- **Governance/policy:** “One Health”, “pandemic preparedness”, “zoonotic disease governance”, “Norway”, “Norwegian Veterinary Institute”, “Mattilsynet”
- **Communication/nursing:** “risk communication”, “health literacy”, “helsesykepleier”, “public health nursing”, “school health service”, “child and family health communication”

### 5. Study-selection criteria

**Included:** Peer-reviewed articles, official institutional risk assessments, surveillance reports, and guidance documents reporting primary epidemiological, virological, or risk-communication evidence relevant to (i) influenza A cross-species transmission mechanisms, (ii) Norwegian or international H5N1 surveillance and epidemiology, (iii) One Health governance structures, or (iv) child- and family-oriented health communication and health-literacy frameworks.

**Excluded:** Conference abstracts without accompanying full text, sources without accessible full text, and non-English-language sources.

**Selection approach:** Purposive rather than exhaustive, prioritizing the most directly relevant and most recent authoritative sources for each concept group, consistent with the narrative mini review format and the Frontiers word-count limit.

### 6. Verification update (reviewer response revision)

As part of responding to reviewer comments, a targeted verification pass was conducted on the reference list and on specific epidemiological claims flagged as highest-risk (exact case-count date ranges, the Los Angeles County cat-to-human serosurvey finding, and author/journal/volume/page details for ten reference entries). This verification used the same database set listed above (primarily PubMed/MEDLINE and direct publisher/journal websites) to confirm publication details against primary sources. Corrections arising from this verification are documented in the accompanying Response to Reviewers letter.

### 7. Limitations

This is a narrative, purposively-selected evidence base appropriate to a Mini Review, not a systematic review. It does not claim database coverage equivalent to a systematic review (e.g., CINAHL, Embase, or Scopus were not searched), dual independent screening was not performed, and no PRISMA flow diagram or formal risk-of-bias assessment was produced. These limitations are stated in the manuscript’s methodology paragraph and are consistent with the scope and word-count constraints of the Mini Review article type.
